# The effect of circulating CD4+ and CD8+ T cells on the prognosis in advanced postoperative pancreatic adenocarcinoma patients who received chemotherapy

**DOI:** 10.1097/MD.0000000000046077

**Published:** 2025-11-21

**Authors:** Qingqing Chang, Hong Fan, Weiwei Si, Jibin Mao

**Affiliations:** a Department of Pathology, Affiliated Hospital 2 of Nantong University, Nantong, Jiangsu Province, China; b Department of Pathology, Jiading District Anting Hospital of Shanghai, Shanghai, China; c Department of Radiation Oncology, The Affiliated Hospital of Nantong University, Nantong, Jiangsu Province, China.

**Keywords:** chemotherapy, circulating immune cells, pancreatic adenocarcinoma, prognosis

## Abstract

The purpose of this study was to analyze the impact of circulating immune cells before and after chemotherapy on the prognosis of patients with advanced pancreatic adenocarcinoma (PAC). Data were collected from 74 advanced PAC patients with post-chemotherapy, retrieved from the Affiliated Hospital of Nantong University database in Jiangsu. Based on the cutoff values of circulating CD4+ and CD8+ T cells before and after chemotherapy, the aforementioned patients were divided into high- and low-level groups. The impact of the expression levels of blood immune cells before and after chemotherapy on patient prognosis was analyzed separately. For both pre- and post-treatment circulating CD4+ and CD8+ T cells, patients in the high expression group had significantly better progression-free survival and overall survival than those in the low expression group (*P* < .001). The results of this study show that high circulating immune cells are positively associated with better prognosis in patients with advanced PAC receiving chemotherapy and may become a new prognostic indicator for PAC treatment.

## 1. Introduction

Pancreatic cancer is one of the deadliest malignant tumors worldwide. In the United States, pancreatic cancer is projected to become the second leading cause of cancer death, following lung cancer, by 2030.^[1]^ The most common pathological type of pancreatic cancer is pancreatic adenocarcinoma (PAC).^[2]^ Additionally, 90% of patients with pancreatic cancer are already in advanced stages at the time of diagnosis.^[2]^ Unfortunately, only 12% of patients with advanced, inoperable pancreatic cancer survive beyond 5 years.^[3]^ Due to the lack of mutations available for targeted therapy, chemotherapy remains the mainstay of treatment for advanced pancreatic cancer.^[4]^

The pancreatic tumor immune microenvironment is defined as the immune environment where the tumor resides, including immune cells, fibroblasts, inflammatory cells, signaling molecules, and the extracellular matrix, all of which are related to the occurrence, development, and metastasis of the tumor.^[5]^ Circulating immune cells and tumor-infiltrating immune cells are simultaneously distinct and dynamically connected.^[6]^ Tumor-infiltrating immune cells originate chiefly from their cognate subsets in the peripheral circulation.^[7]^ Guided by chemokines, cytokines, and endothelial signals, these circulating cells traverse the vascular wall and enter the tumor, thereby becoming tumor-infiltrating immune cells.^[8]^ Pancreatic cancer is classified as an immunological desert tumor due to the relative scarcity of effector T cells infiltrating within and around the tumor.^[9]^ Additionally, there is a higher presence of cells that suppress immune function within the pancreatic cancer tissue, such as immunosuppressive Tregs, MDSCs, and TAMs of the immunosuppressive phenotype (M2).^[10]^ Furthermore, pancreatic cancer cells can produce immunosuppressive cytokines, including IL-10, IL-1β, TGF-β, etc. Consequently, pancreatic cancer is less responsive to immunotherapy.^[11]^ Chronic pancreatitis, marked by persistent inflammation, progressive fibrosis and irreversible loss of exocrine and endocrine function, is a well-established independent risk factor for pancreatic cancer.^[12]^ Concurrently, autoimmune pancreatitis displays a complex and multifaceted association with pancreatic malignancy.^[13]^

In recent years, immunotherapy has made revolutionary progress in the treatment of solid malignant tumors. However, the effectiveness of immunotherapy on pancreatic cancer has been minimal, with many clinical studies failing to yield significant results. At the same time, current research on immune cells in pancreatic cancer is focused on infiltrating immune cells, while the study of circulating immune cells in serum has not received adequate attention. A deeper understanding of the immune microenvironment and circulating immune cells in advanced pancreatic cancer could be beneficial for the further development of new therapeutic approaches and prognostic indicators.

## 2. Patients and methods

### 2.1. Study design and patient selection

This retrospective clinical cohort study was conducted to assess the prognostic value of circulating CD4+ and CD8+ T cell subsets in patients with advanced resected PAC receiving adjuvant chemotherapy. The authors searched the internal database from the Affiliated Hospital of Nantong University (Nantong, China) with a hospital’s medical records browsing protocol. The medical records confirm that the patient was diagnosed with advanced PAC and subsequently underwent at least 2 rounds of standard chemotherapy. In this study, the staging of PAC is based on the eighth edition of the American Joint Committee on Cancer Staging Manual TNM staging.^[14]^ The following content outlines the detailed inclusion criteria for this study: Pancreatic adenocarcinoma with distant metastasis was confirmed by pathology and imaging between January 2020 and May 2021 with a hospital’s medical records; The patients were treated with chemotherapy after the initial diagnosis for 2 cycles; Patients were given maintenance therapy until the tumor progressed or death; and Circulating immune cells were detected before and after 2 cycles of chemotherapy. Additionally, the exclusion criteria for this study are as follows: Patients who did not receive chemotherapy for various reasons after confirmation of diagnosis; Incomplete medical records and clinical information; and Patients who cannot cooperate with follow-up work. Through the aforementioned inclusion criteria, this study ultimately enrolled 74 patients with advanced PAC. The aforementioned patients were divided into different groups based on the levels of circulating immune cells (CD4+ and CD8+) before and after chemotherapy.

### 2.2. Data collection

This study retrieved patients’ clinical information from medical records, including gender, age, performance status (PS), smoking status, albumin, tumor differentiation, metastatic site, tumor markers, chemotherapy, and circulating immune cells. The authors carefully reviewed the medical records of each patient and meticulously documented their clinical information and significant data. The clinical information and tumor data of each patient post-diagnosis and pretreatment were recorded as baseline data and analyzed. The authors collected and statistically analyzed the data on circulating immune cells before and after chemotherapy for the patients, focusing primarily on CD4+ and CD8+ T cells. Peripheral blood CD4+ and CD8+ T cell quantification was performed at the Affiliated Hospital of Nantong University using a four-color BD FACSCanto II flow cytometer (BD Biosciences, San Jose) equipped with FACSCanto clinical software (v3.0). Fresh whole-blood samples (100 µL) were stained for 15 minutes at room temperature with the following fluorochrome-conjugated monoclonal antibody cocktail: CD4-APC (clone SK3) and CD8-PerCP (clone SK1) (all from BD Biosciences). After red-cell lysis with BD FACS Lysing Solution (10 minutes, room temperature, in the dark), cells were washed and immediately acquired. Absolute counts (cells/µL) were calculated using BD TruCount tubes according to the manufacturer’s instructions. The antibodies used in the detection of circulating immune cells were CD4-FITC and CD8-PE (BCS Company, Inc., BD Biosciences lnc., San Jose).

### 2.3. Immune cell-related survival analysis by database

Immune cell-related survival analysis was performed using TIMER.^[15]^ TIMER is an interactive cancer database for researching immune cell infiltration. The prognostic analysis functions of TIMER were utilized to analyze the impact of immune cells on the prognosis in PAC with different immune cell levels. A *P*-value < .05 represents a significant impact on prognosis.

### 2.4. Response, survival evaluation

According to the response evaluation criteria in solid tumors, 2 radiologists calculated the objective response of tumors independently from different groups after 2 cycles of chemotherapy.^[16]^ In this study, every patient underwent systematic imaging examinations after 2 cycles of chemotherapy. A partial response (PR) was defined as a ≥30% decrease in the sum of the longest diameter for all target lesions. Progressive disease was defined as either an increase of ≥20% in the sum of the longest diameters of all target lesions or the appearance of new tumor lesions. The condition that is intermediate between these 2 states is defined as stable disease (SD). The authors made a statistical analysis of progression-free survival (PFS) and overall survival (OS) time of each patient in different groups. PFS was defined as the time from the beginning of treatment to the progression of disease or death. OS was defined as the time from the beginning of randomization to death for any reason.^[17,18]^

### 2.5. Statistical analyses

The χ^2^ test was used to analyze the patient characteristics of different groups. Continuous variables were estimated using the unpaired Student *t* test. Cox multivariate analysis was used to evaluate the influence of clinical variables on prognosis. The Cox multivariate model included important confounding variables such as gender, age, PS, smoking status, albumin, tumor differentiation, metastatic site, tumor markers, chemotherapy, and circulating immune cells. The differences in survival between the 2 groups were compared using the Kaplan–Meier method and Log-rank test. Data analysis was done by SPSS version 17.0 (SPSS, Inc., Chicago). *P* < .05 was considered statistically significant. The line-fitting method was used to investigate the correlation between the numbers of circulating immune cells before and after chemotherapy in patients. Linear correlation can be judged by the value of R2. The closer the value of R2 is to 1, the higher the linear correlation between the 2 sets of data. An R2 value above 0.7 indicates a significant correlation.^[19]^ The analysis was performed using GraphPad Prism version 8.2 (GraphPad Software, Inc., San Diego).

### 2.6. Ethical statement

This study was approved by The Institutional Review Board of the Affiliated Hospital of Nantong University (Nantong, China; 2023; IRB no.: JSNT20230149). All participants provided written informed consent.

## 3. Results

### 3.1. Association of immune cells with survival by database analysis

**Figure 1.**
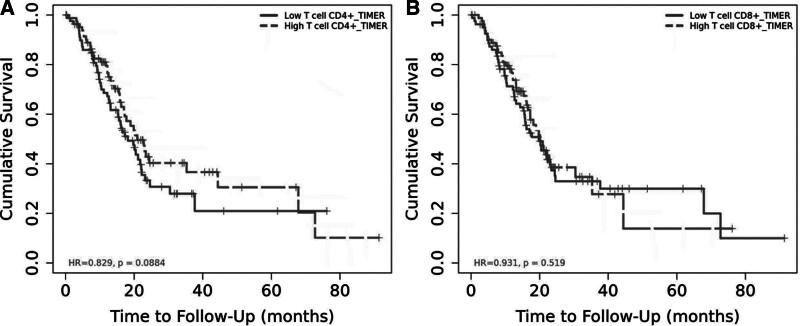
Database analysis on the impact of immune cells on the survival of PAC. PAC = pancreatic adenocarcinoma.

The analysis of the impact of immune cells on the cumulative survival of PAC patients was conducted by TIMER database. For T cell series, our research showed no significant impact neither CD4+ T cells (hazard ratio (HR) = 0.829, *P* = .088), nor CD8+ T cells (HR = 0.931, *P* = .519) have on the OS (Fig. 1).

### 3.2. Patient characteristics

The authors initially enrolled and collected medical records of 292 patients with PAC. Among them, 156 patients were excluded due to incomplete medical records or lack of immune cell data. Forty-two patients were excluded because they could not tolerate at least 2 cycles of chemotherapy. Nineteen patients were excluded as they were lost to follow-up. One patient was excluded due to the presence of another type of malignant tumor. In the end, a total of 74 patients with advanced PAC who met the inclusion criteria were included in this study (Tables 1 and 2). All patients had distant metastases, with 49 presenting liver metastases and the remaining 25 involving other sites: 18 in the lungs and 7 in distant lymph nodes. All patients received 2 cycles of chemotherapy after diagnosis and continued with maintenance treatment until the study’s observation endpoint. Circulating lymphocyte levels for each group are summarized below: pretreatment CD4+ T cells: high (n = 36), low (n = 38); post-treatment CD4+ T cells: high (n = 32), low (n = 42); pretreatment CD8+ T cells: high (n = 36), low (n = 38); post-treatment CD8+ T cells: high (n = 32), low (n = 42).

**Table 1:** Patients demographics and study treatment by CD4+ T cell.

Patient characteristics	High pretreatment CD4+ T cell (n = 36)	Low pretreatment CD4+ T cell (n = 38)	*P*-value	High posttreatment CD4+ T cell (n = 32)	Low posttreatment CD4+ T cell (n = 42)	*P*-value
Sex, n (%)						
Male	19 (52.8%)	21 (55.3%)	.831	17 (53.1%)	23 (54.8%)	.892
Female	17 (47.2%)	17 (44.7%)		15 (46.9%)	19 (45.2%)	
Median age, yr (range)	68.5 (38–88)	67.5 (33–84)	.813	68.5 (38–88)	67.5 (33–84)	.764
Smoking status, n (%)						
Never-smoker	21 (58.3%)	21 (55.3%)	.788	18 (56.2%)	24 (57.1%)	.946
Former/current smoker	15 (41.7%)	17 (44.7%)		14 (43.8%)	18 (42.9%)	
Mean albumin, g/L (SD)	40.2 (5.5)	39.5 (4.9)		39.6 (5.2)	40.0 (5.2)	
Performance status, n (%)						
0–1	29 (80.6%)	26 (68.4%)	.232	27 (84.4%)	28 (66.7%)	.082
>1	7 (19.4%)	12 (31.6%)		5 (15.6%)	14 (33.3%)	
Tumor differentiation						
Poor	17 (47.2%)	15 (39.5%)	.495	14 (43.8%)	18 (42.9%)	.938
Moderate	19 (52.8%)	23 (60.5%)		18 (56.2%)	24 (57.1%)	
Metastases, n (%)						
Liver metastases	27 (75.0%)	22 (57.9%)	.124	24 (75.0%)	25 (59.5%)	.162
Others	9 (25.0%)	16 (42.1%)		8 (25.0%)	17 (40.5%)	
Mean CA 19-9, U/mL (SD)	7306.1 (4838.3)	8026.9 (4347.0)	.189	7307.2 (4781.4)	7957.4 (4449.3)	.331
Mean CEA, ng/mL (SD)	18.1 (9.9)	21.6 (13.0)	.233	18.9 (10.2)	20.7 (12.7)	.309
Chemotheray, n (%)						
TAX/ABX + GEM	25 (69.4%)	26 (68.4%)	.925	21 (65.6%)	30 (71.4%)	.587
Others	11 (30.6%)	12 (31.6%)		11 (34.4%)	12 (28.6%)	

χ^2^ test was used to compare patient characteristics, and *P*-value < .05 was statistically significant.

ABX = albumin-bound paclitaxel, CA 19-9 = cancer antigen 19-9, CEA = carcinoembryonic antigen, GEM = gemcitabine, SD = standard deviation, TAX = paclitaxel.

**Table 2:** Patients demographics and study treatment by CD8+ T cell.

Patient characteristics	High pretreatment CD8+ T cell (n = 35)	Low pretreatment CD8+ T cell (n = 39)	*P*-value	High posttreatment CD8+ T cell (n = 39)	Low posttreatment CD8+ T cell (n = 35)	*P*-value
Sex, n (%)						
Male	18 (51.4%)	22 (56.4%)	.672	20 (51.3%)	20 (57.1%)	.614
Female	17 (48.6%)	17 (43.6%)		19 (48.7%)	15 (42.9%)	
Median age, yr (range)	68 (38–88)	68 (33–84)	.774	69 (38–88)	68 (33–84)	.725
Smoking status, n (%)						
Never-smoker	22 (62.9%)	20 (51.3%)	.318	23 (59.0%)	19 (54.3%)	.688
Former/current smoker	13 (37.1%)	19 (48.7%)		16 (41.0%)	16 (45.7%)	
Mean albumin, g/l (SD)	39.1 (5.9)	40.2 (4.6)		39.2 (5.8)	40.5 (4.4)	
Performance status, n (%)						
0–1	28 (80.0%)	27 (69.2%)	.292	30 (76.9%)	25 (71.4%)	.591
>1	7 (20.0%)	12 (30.8%)		9 (23.1%)	10 (28.6%)	
Tumor differentiation						
Poor	14 (40.0%)	18 (46.2%)	.587	16 (41.0%)	16 (45.7%)	.683
Moderate	21 (60.0%)	21 (53.8%)		23 (59.0%)	19 (54.3%)	
Metastases, n (%)						
Liver metastases	24 (68.6%)	25 (64.1%)	.689	25 (64.1%)	24 (68.6%)	.694
Others	11 (31.4%)	14 (35.9%)		14 (35.9%)	11 (31.4%)	
Mean CA 19-9, U/mL (SD)	7172.6 (4693.2)	7900.1 (4633.2)	.172	7063.1 (4579.3)	8359.5 (4538.1)	.097
Mean CEA, ng/mL (SD)	17.7 (10.1)	21.8 (12.8)	.159	17.3 (9.6)	22.7 (13.1)	.152
Chemotherapy, n (%)						
TAX/ABX + GEM	22 (62.9%)	29 (74.4%)	.292	25 (64.1%)	26 (74.3%)	.359
Others	13 (37.1%)	10 (25.6%)		14 (35.9%)	9 (25.7%)	

χ^2^ test was used to compare patient characteristics, and *P*-value < .05 was statistically significant.

ABX = albumin-bound paclitaxel, CA 19-9 = cancer antigen 19-9, CEA = carcinoembryonic antigen, GEM = gemcitabine, SD = standard deviation, TAX = paclitaxel.

**Figure 2.**
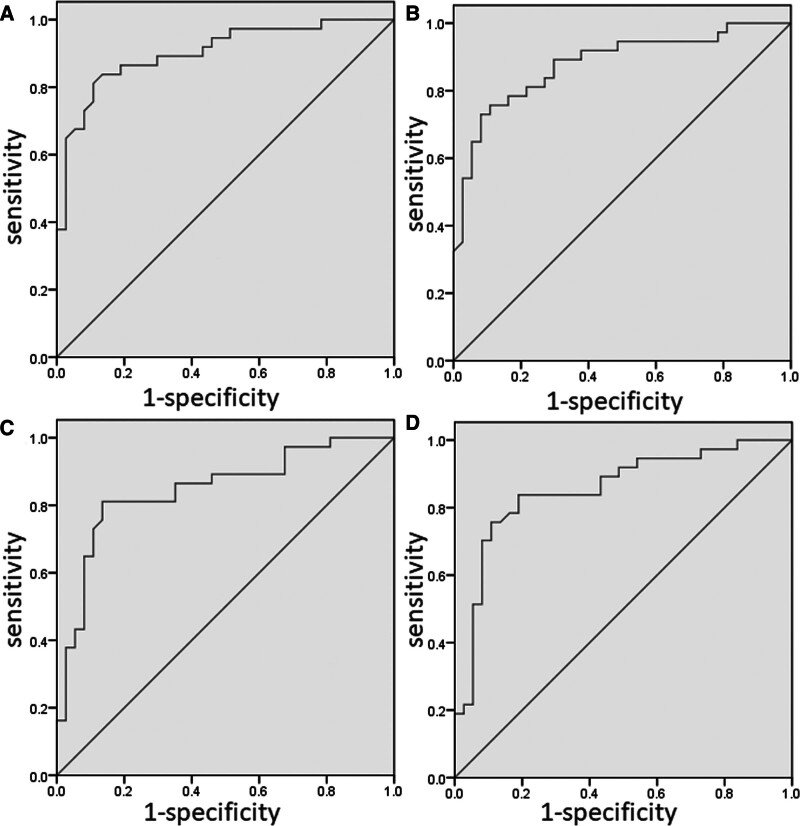
Cutoff value analysis for circulating immune cells.

Based on the survival data analysis of the patients included in this article, the cutoff values for pretreatment and posttreatment CD4+ T cells are 608/μL and 666/μL, respectively. The cutoff values for pretreatment and posttreatment CD8+ T cells are 332/μL and 377/μL, respectively (Fig. 2). Based on the cutoff values for immune cells, the 74 patients were divided into 8 groups, and comparisons were made between the corresponding pairs of groups. The data analysis revealed that among the 2 groups of patients being compared, there were no significant statistical differences in factors such as gender, age, PS, smoking status, albumin, tumor differentiation, metastatic site, tumor markers, and chemotherapy (all *P* > .05). This indicates that the clinical data among the groups of patients are well balanced (Tables 1 and 2).

### 3.3. Survival analysis of pretreatment circulating serum immune cells

**Figure 3.**
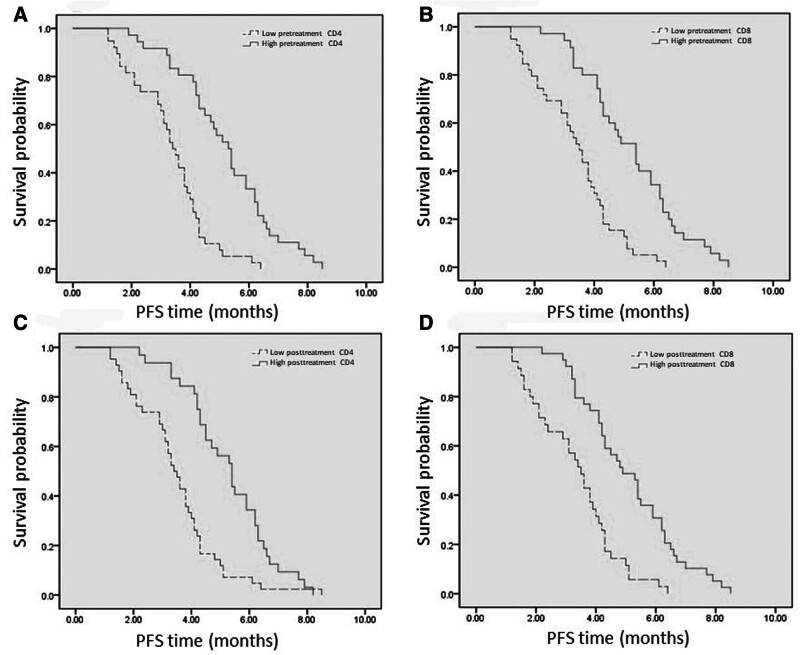
Survival analysis of posttreatment circulating immune cells.

The survival of patients with different levels of circulating immune cells before treatment is as follows: Patients in the low pretreatment CD4+ T cell group had a median progression-free survival (mPFS) of 3.5 months and a median overall survival (mOS) of 10.8 months. Patients in the high pretreatment CD4+ T cell group had a mPFS of 5.3 months and a mOS of 13.2 months. Patients in the low pretreatment CD8+ T cell group had a mPFS of 3.4 months and a mOS of 10.6 months. In contrast, patients in the high pretreatment CD8+ T cell group had a mPFS of 5.4 months and a mOS of 12.9 months. Compared to the low pretreatment CD4+ T cell group, patients in the high CD4+ T cell group also exhibited better PFS (adjusted HR, 0.177; 95% CI: 0.098–0.338; *P* < .001) and OS (adjusted HR, 0.069; 95% CI: 0.030–0.157; *P* < .001). Similarly, patients in the high pretreatment CD8+ T cell group had superior survival times in terms of both PFS (adjusted HR, 0.171; 95% CI: 0.087–0.335; *P* < .001) and OS (adjusted HR, 0.147; 95% CI: 0.077–0.280; *P* < .001) compared to the low CD8+ T cell group. These data were derived from multivariate COX regression analysis (Fig. 3).

### 3.4. Survival analysis of posttreatment circulating immune cells

**Figure 4.**
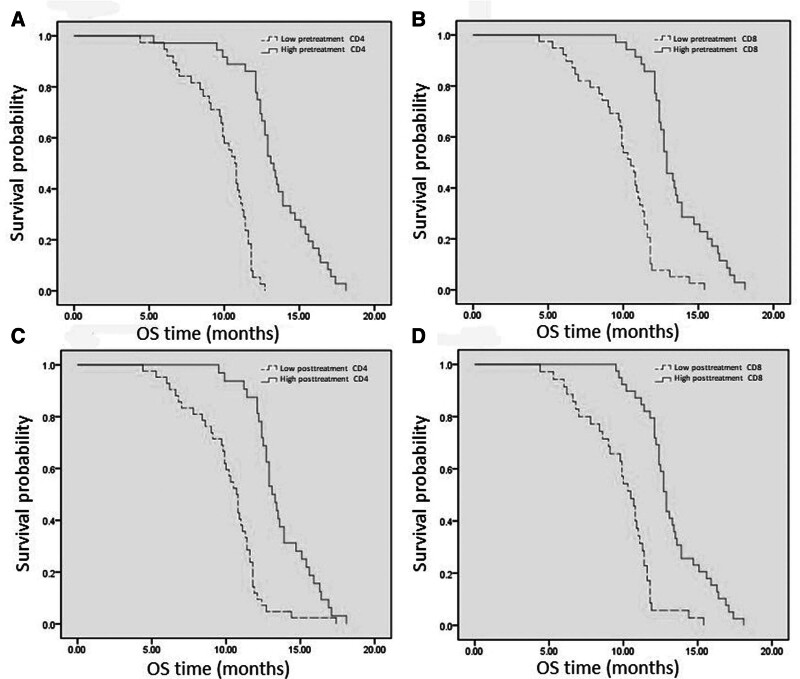
Survival analysis of pretreatment circulating immune cells.

The survival outcomes of patients with different levels of circulating immune cells after treatment are as follows: Patients in the low post-treatment CD4+ T cell group had a mPFS of 3.4 months and a mOS of 10.7 months. Patients in the high post-treatment CD4+ T cell group had a mPFS of 5.4 months and a mOS of 13.2 months. Patients in the low post-treatment CD8+ T cell group had a mPFS of 3.5 months and a mOS of 10.5 months. Patients in the high post-treatment CD8+ T cell group had a mPFS of 4.9 months and a mOS of 12.8 months. Compared to the low CD4+ T cell group, patients in the high post-treatment CD4+ T cell group also had better PFS (adjusted HR, 0.210; 95% CI: 0.112–0.393; *P* < .001) and OS (adjusted HR, 0.145; 95% CI: 0.077–0.237; *P* < .001). Similarly, patients in the high post-treatment CD8+ T cell group had superior survival times in PFS (adjusted HR, 0.257; 95% CI: 0.144–0.458; *P* < .001) and OS (adjusted HR, 0.167; 95% CI: 0.091–0.306; *P* < .001) compared to the low CD8+ T cell group. All of the above results took into account significant clinical factors in the analysis (Fig. 4).

### 3.5. Subgroup analysis of overall survival

**Figure 5.**
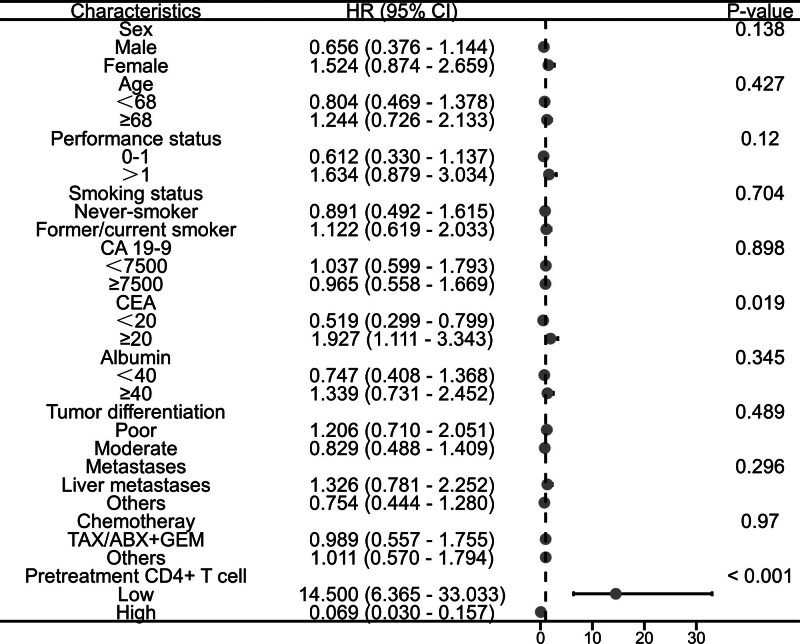
Subgroup analysis of overall survival based on pretreatment CD4+ T cell level.

**Figure 6.**
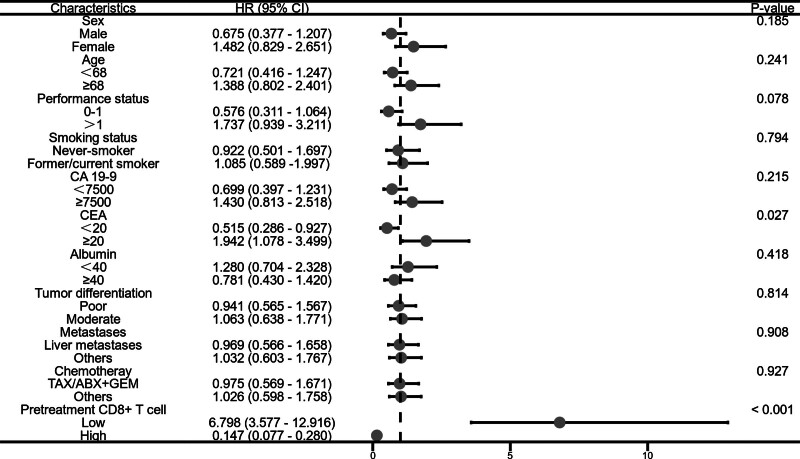
Subgroup analysis of overall survival based on pretreatment CD8+ T cell level.

**Figure 7.**
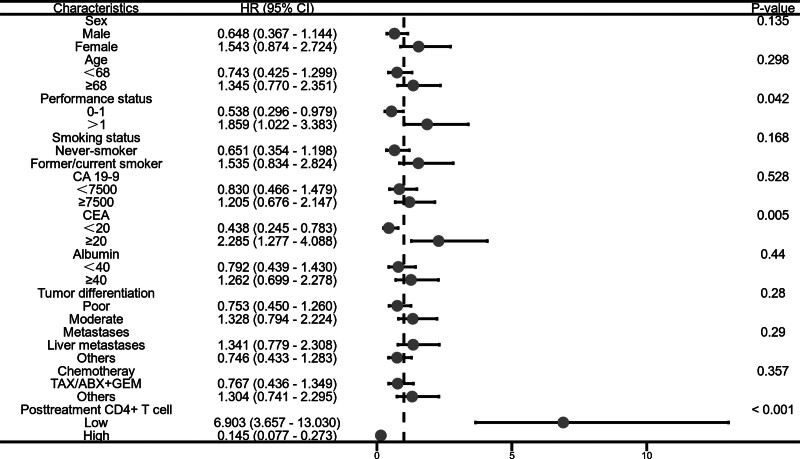
Subgroup analysis of overall survival based on posttreatment CD4+ T cell level.

**Figure 8.**
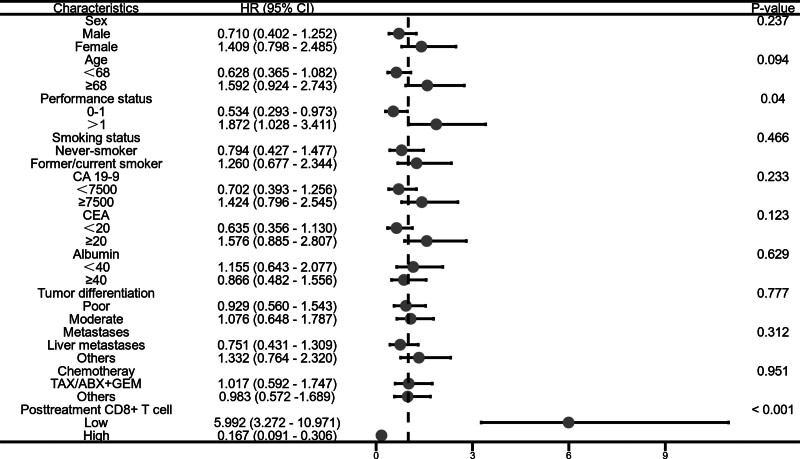
Subgroup analysis of overall survival based on posttreatment CD8+ T cell level.

This study conducted further subgroup analysis on the patients’ survival data. The data analysis of pretreatment CD4+ T cell stratification showed that the OS of patients with low carcinoembryonic antigen (CEA) levels was significantly lower than that of the high CEA group (adjusted HR, 0.519; 95% CI: 0.299–0.799; *P* = .019) (Fig. 5). Similarly, the analysis of pretreatment CD8+ T cell data indicated that the OS of patients with low CEA levels was significantly lower than that of the high CEA group (adjusted HR, 0.515; 95% CI: 0.286–0.927; *P* = .027) (Fig. 6). The analysis of post-treatment CD4+ T cell data revealed that the OS of patients with low CEA levels was significantly lower than that of the high CEA group (adjusted HR, 0.438; 95% CI: 0.245–0.783; *P* = .005). Additionally, the OS of patients in the low PS group was significantly shorter than that of the high PS group (adjusted HR, 0.583; 95% CI: 0.296–0.979; *P* = .042) (Fig. 7). Likewise, the analysis of post-treatment CD8+ T cell data showed that the OS of patients with low PS was significantly shorter than that of the high PS group (adjusted HR, 0.635; 95% CI: 0.356–1.130; *P* = .027) (Fig. 8). No significant statistical differences were observed among the other relevant subgroups.

### 3.6. Response to treatment by circulating immune cells

This study analyzed the impact of the quantity of different immune cells at various stages on patients’ responses to chemotherapy. The proportion of progressive disease (PD) in patients with a high pre-chemotherapy CD4+ T cell group was significantly lower than that in the high CD4+ T cell group (*P* = .007). The same result was observed in the analysis of CD4+ T cells after chemotherapy, where the rate of PD in the high-level group was significantly lower than in the low-level group (*P* = .005). However, whether before or after chemotherapy, the level of CD4+ T cells did not have a significant effect on the rates of PR and SD (all *P* values > .05) (Table 3).

**Table 3:** Response to treatment by serum CD4+ T cell.

Treatment response	High pretreatment CD4+ T cell (n = 36)	Low pretreatment CD4+ T cell (n = 38)	*P*-value	High posttreatment CD4+ T cell (n = 32)	Low posttreatment CD4+ T cell (n = 42)	*P*-value
Partial response, n (%)	4 (11.1%)	1 (2.6%)	.149	4 (12.5%)	1 (2.4%)	.086
Stable disease, n (%)	29 (80.6%)	24 (63.2%)	.096	26 (81.3%)	27 (64.3%)	.109
Progressive disease, n (%)	3 (8.3%)	13 (34.2%)	.007	2 (6.2%)	14 (33.3%)	.005

After receiving chemotherapy, patients in the high pretreatment circulating CD8+ T cell group had a higher rate of SD (*P* = .042) and a lower rate of PD (*P* = .009) compared to those in the lower-level group. Furthermore, compared to the post-chemotherapy low circulating CD8+ T cell group, the high circulating CD8+ T cell group had a greater number of patients achieving SD (*P* = .036) and fewer patients experiencing PD (*P* = .002). However, the impact of circulating CD8+ T cell levels on the rate of PR in patients was not significant (all *P* values > .05) (Table 4).

**Table 4:** Response to treatment by serum CD8+ T cell.

Treatment response	High pretreatment CD8+ T cell (n = 35)	Low pretreatment CD8+ T cell (n = 39)	*P*-value	High posttreatment CD8+ T cell (n = 39)	Low posttreatment CD8+ T cell (n = 35)	*P*-value
Partial response, n (%)	4 (11.4%)	1 (2.6%)	.129	4 (10.2%)	1 (2.9%)	.205
Stable disease, n (%)	29 (82.9%)	24 (61.5%)	.042	32 (82.1%)	21 (60.0%)	.036
Progressive disease, n (%)	2 (5.7%)	14 (35.9%)	.009	3 (7.7%)	13 (37.1%)	.002

### 3.7. Correlation analysis of circulating immune cells

**Figure 9.**
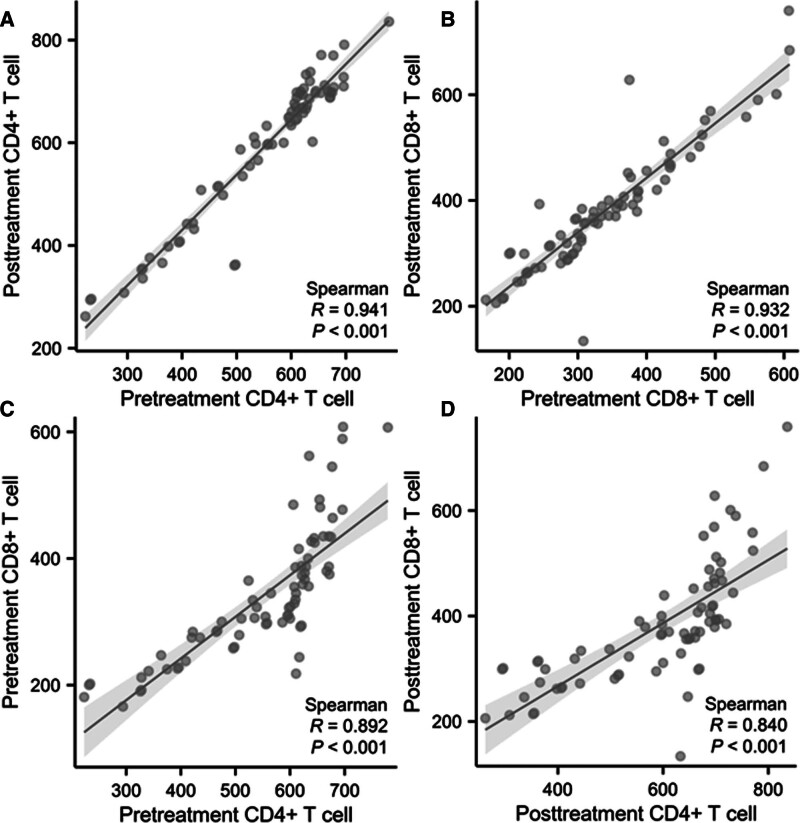
Correlation analysis between circulating immune cells.

This study analyzed the correlation of circulating immune cells before and after chemotherapy in PAC patients. The results showed a positive correlation in the number of CD4+ T cells before and after chemotherapy (*R* = 0.941, *P* < .001), and a similar finding was observed for CD8+ T cells before and after chemotherapy (*R* = 0.932, *P* < .001). Subsequently, we analyzed the correlation between the numbers of CD4+ and CD8+ T cells before chemotherapy and found that they were positively correlated (*R* = 0.892, *P* < .001). Furthermore, there is also a positive correlation between the numbers of CD4+ and CD8+ T cells after chemotherapy (*R* = 0.840, *P* < .001) (Fig. 9).

## 4. Discussion

To our knowledge, this study is the first to demonstrate that high circulating CD4+ and CD8+ T cell counts before and after chemotherapy are associated with better prognosis in advanced PAC. These findings highlight circulating CD4+ and CD8+ T cells as potential prognostic markers for this disease.

We retrospectively reviewed 74 patients with advanced PAC who received postoperative chemotherapy and analyzed their circulating immune cells at both time points. Subgroup analyses confirmed that elevated CD4+ and CD8+ T cell levels before and after treatment correlated closely with improved survival. Correlation analysis further revealed a strong association between pre- and post-treatment CD4+ and CD8+ T cell counts.

Collectively, our data clearly show that maintaining high circulating CD4+ and CD8+ T cell levels is linked to favorable outcomes in advanced PAC. This underscores the value of these immune subsets as readily accessible prognostic indicators and supports their integration into clinical monitoring strategies.

Circulating immune cells play transformative, replenishing, and regulatory roles in shaping tumor-infiltrating immune cells; consequently, they are pivotal for tumor control and therapy. Pancreatic cancer is a type of tumor that is highly immunosuppressive.^[20]^ This immunosuppressive environment has a significant impact on the development and treatment of advanced pancreatic cancer. The tumor microenvironment creates an immunosuppressive milieu by triggering mechanisms that promote immune evasion and limit the activation of effective anti-tumor immune responses.^[21]^ The immune microenvironment of pancreatic cancer is a complex system composed of a variety of cells and factors.^[22]^

In the context of pancreatic cancer, immune checkpoint inhibitors (ICIs) have shown some promise, particularly in combination with other treatments.^[23]^ For instance, the combination of the PD-1 inhibitor pembrolizumab with chemotherapy has been approved by the U.S. Food and Drug Administration for the treatment of patients with metastatic pancreatic cancer who have progressed after standard chemotherapy.^[24]^

However, the effectiveness of ICIs in pancreatic cancer remains limited. Pancreatic tumors often have a high degree of immunosuppression and a dense stroma that can hinder T-cell infiltration, making them less responsive to ICIs.^[25]^ Additionally, the use of ICIs can be associated with immune-related adverse events, which can range from mild to severe and life-threatening.^[26]^ Researchers are actively exploring strategies to enhance the effectiveness of ICIs in pancreatic cancer, such as combining them with other immunotherapies, using them in earlier stages of the disease, or identifying biomarkers to predict which patients are most likely to benefit from this type of treatment.^[27,28]^

Of course, there are some shortcomings in this study as well. First, although the study utilized multifactor analysis, retrospective clinical research inevitably has certain biases. Secondly, the strict inclusion criteria of this study led to a smaller number of cases being included, which may result in less accurate research outcomes. Additionally, the cases of advanced PAC patients included in this study have regional limitations. Lastly, the study only revealed the relationship between circulating immune cells and patient prognosis, but did not explore the deeper molecular mechanisms. Therefore, further research is needed for us to design the next phase of the study. The results of this study are far from clinical application. Therefore, the results obtained in this study need to be interpreted and applied with caution.

## 5. Conclusion

This study reveals that for PAC undergoing chemotherapy, higher levels of circulating immune cells before and after chemotherapy are associated with better treatment responses and prognosis. Moreover, there is a positive correlation between circulating CD4+ and CD8+ T cells.

## Acknowledgments

The author of this study should sincerely thank Dr Feng Zhao (Department of Medical Records, The Affiliated Hospital of Nantong University) for providing and maintaining the clinical database for the professional data analysis guidance.

## Author contributions

**Conceptualization:** Qingqing Chang, Hong Fan.

**Data curation:** Qingqing Chang, Hong Fan, Weiwei Si, Jibin Mao.

**Formal analysis:** Qingqing Chang, Hong Fan, Weiwei Si, Jibin Mao.

**Methodology:** Weiwei Si.

**Supervision:** Weiwei Si, Jibin Mao.

**Validation:** Weiwei Si.

**Writing – original draft:** Qingqing Chang, Hong Fan.

**Writing – review & editing:** Qingqing Chang, Jibin Mao.
